# Understanding the Reasons Why Men and Women Do Not Donate Gametes

**DOI:** 10.1007/s43032-022-01112-9

**Published:** 2022-11-18

**Authors:** Stephen Whyte, Ho Fai Chan, Nikita Ferguson, Megan Godwin, Karin Hammarberg, Benno Torgler

**Affiliations:** 1 School of Economics and Finance, Queensland University of Technology, Gardens Point, 2 George St, Brisbane, QLD 4001 Australia; 2 Centre in Regenerative Medicine, Institute of Health and Biomedical Innovation, Queensland University of Technology, 60 Musk Avenue, Kelvin Grove, QLD 4059 Australia; 3 Centre for Behavioural Economics, Society and Technology, Queensland University of Technology, Brisbane, QLD 4000 Australia; 4 School of Advertising, Marketing & Public Relations, Queensland University of Technology, Gardens Point, 2 George St, Brisbane, QLD 4001 Australia; 5 School of Public Health and Preventive Medicine, Monash University, 549 St Kilda Rd, Melbourne, Victoria 3004 Australia; 6 CREMA—Center for Research in Economics, Management and the Arts, Zurich, Switzerland

**Keywords:** Donation, Sperm, Oocyte, Sex difference, Willingness, Barriers

## Abstract

The global under-supply of sperm and oocyte donors is a serious concern for assisted reproductive medicine. Research has explored self-selected populations of gamete donors and their ex-post rationalisations of why they chose to donate. However, such studies may not provide the necessary insight into why the majority of people do not donate. Utilising the unique open form responses of a large sample (*n* = 1035) of online survey respondents, we examine the reasons participants cite when asked: “*Why haven’t you donated your sperm/eggs?*.” We categorise these responses into four core themes (*conditional willingness*, *barriers*, *unconsidered*, and *conscientious objector*) and eleven lower-order themes. We find that, on average, women are more conditionally willing (8.2% difference; *p* = 0.008) to participate in gamete donation than men. We also find that women are more likely than men to justify their non-donation based on their reproductive history (21.3% difference; *p* = 0.000) or kin selection and inclusive fitness (5.7% difference; *p* = 0.008). However, compared to women, men are more likely to validate their non-donation based on sociocultural or social norms (6% difference; *p* = 0.000) or religion (1.7% difference; *p* = 0.030). That so many of our study participants report in-principal willingness for future participation in gamete donation speaks to the need for increased research on understanding non-donor population preferences, motivations, and behaviours.

## Introduction

The demand for donated gametes for use in assisted reproductive medicine outstrips supply in most countries [1–7]. Since the advent of assisted reproductive technologies (ART) in the late twentieth century, the need for gametes has grown exponentially. Across this time, many social science disciplines have sought to understand what motivates men and women to donate their gametes to other individuals, as well as to commercial ART organisations and science [2, 8–21]. However, previous research has almost exclusively focused on donors’ and their ex-post rationalisation of their decision-making process. While such studies inform an understanding of both positive and negative correlates of donation behaviour and its possible drivers, they provide little insight into the preferences, experiences, understanding, and decision processes of those yet to donate.

Arguably, the most effective method for increasing gamete donation is to secure *new* donors. Behavioural research can be helpful in this effort by exploring the non-donor population’s preferences and behaviour. As such, our current study deliberately targets non-donors, inviting them to respond openly(in writing) to the broader question of why they have not yet donated. In a population of non-donors, we asked over 1000 online survey respondents: “*Why haven’t you donated your sperm/eggs?*.” Our study seeks to identify recurring motivational themes and barriers to gamete donation in a non-donor population and also to explore any sex differences associated with such.

### Background

Gamete donation research across a range of scientific disciplines has explored and identified key globally recurring motivations for donation. Firstly, “altruism”—both as an ideological imperative and as a clinical and regulatory practice—appears almost universally throughout the research literature [8, 10, 12–14, 19, 22–24]. Financial remuneration has also been a core focus of gamete donation research. As a procedural method but also as an economic incentivising instrument for participation [1, 2, 8, 12, 13, 16, 22–24]. Donor anonymity has been another key focus of behavioural research as both a barrier and motivation for donation. This is because, historically, donor legislation and the broader ART profession endorsed anonymity and secrecy [25]. Following shifts from anonymity towards openness in donor legislation in several high-income countries, research moved to explore both the preference for and impact of donor identifiability—both at the time of donation and when offspring reach legal age [1, 9, 11, 15, 26–28]. Identifiability of course raises questions regarding donors’ motivation for knowledge of the outcomes of their donations [8, 11, 13] and any direct or indirect influence from a donor’s spouse, partner, and family members when choosing to donate or after having donated [2, 11, 28–30]. More recently (coupled with the advent of the internet as a conduit for human communication, cooperation, and mate choice),research has begun to explore donor and recipient behaviour in unregulated online markets for gamete donation [6, 30–37], as well as the role and impact of new at-home genetic testing and the legal and ethical questions it raises for donors, recipients, and the ART industry [38].

As such, gamete donation research is more necessary and relevant than ever before and is arguably becoming more complex and nuanced in its analysis of both human psychology and donor behaviour. For example, selling gametes (i.e. commercial gamete donation) in Australia is not permitted. However, gifting gametes (altruistic gamete donation) is allowed, provided the donor agrees that information about him or her can be released to any person born as a result of his or her donation [39]. In light of the growing need for increased ART gamete supply, our study seeks to build on previous donation research by exploring non-donor preferences and behaviour in the context of gamete donation.

## Materials and Methods

### Data Collection

This study utilises data from an anonymous online survey, exploring individuals’ reasons for not being a gamete donor. Data were collected between 13 July and 7 September 2017 using the Queensland University of Technology KeySurvey software. Some data from this survey were also analysed in a previous unrelated study [40].

We designed a short survey and asked the participants (*n* = 1035) whether they had previously donated sperm/eggs, depending on the participants’ sex. The question was followed by a free-response question relating to their (non-) participation in RT donation: “*Please provide or explain your feelings or reasons in regard to why you [have/have never] donated your [sperm/eggs]?*”). In addition to sex, the survey also captures information about participants’ age, education level, income level, religious affiliation, and marital status that were used in the analysis of this study. As standard practice for behavioural science research [40], the survey also includes other demographic and socioeconomic questions such as sexuality, political views, and subjective well-being.

The survey was advertised via social media (Facebook) and was open to participants aged 18 years and older who were currently residing in Australia. Participation was incentivised with the chance to win one of four prize draws (lotteries) valued at AUD $250 each. The participants needed to opt-in for the prize draw by entering their email addresses. Email addresses were collected separately and not linked to survey responses. The research was conducted in accordance with the approved Queensland University of Technology (QUT) Human Research Ethics Committee protocol (approval no. 1700000421). Our study meets the relevant COREQ criteria for both (domain 2) study design and (domain 3) analysis [41].

### Participant Characteristics

Our sample consisted of *n* = 1027 participants (681 female and 346 male subjects) who stated they had never previously donated their gametes. Further two (2) female and six (6) male respondents indicated they had previously donated their gametes. As the methodology and data capture of the study solely sought non-gamete donor responses, and as the ratio of donors to non-donors was 0.0078% of the sample population, the eight participants with a gamete donation history were excluded from the study.

Only the participants who provided a response to all the survey questions (complete responses), including those who selected “I prefer not to answer” for specific questions, were included in the analyses. That is, those who did not provide a response to one or more questions (partial completions) were not included in analyses as part of the requirement of the university research ethics committee approval.

The mean age of the participants was 28 years (*SD* = 11.35). The ages ranged between 18 and 70 years. As the study asked the participants to reflect and respond on why they had not donated, the participants beyond reproductive age were included. This was for two reasons. Firstly, it is inequitable to provide an arbitrary chronological age for which both men and women cease to be of reproductive age. Secondly, as the study focused on non-donor self-reflections, the older participants’ perspectives are just as valid data for analysis as those younger participants in current or peak reproductive age.

Most participants (64.37%) reported having post-secondary education (this includes diploma, technical college, and undergraduate and postgraduate university education); 34.27% had completed high school (i.e. to a grade12 level); and the remaining 1.36% had not completed high school.

Most participants (75.07%) reported earning AUD$40,000 or less per annum, while 18.5% reported earning between AUD$40,001 and $200,000. Some participants (5.3%) chose not to provide income information.

Most of the participants (41.09%) reported being an atheist, while 29.5% identified as Christian. Other religious affiliations included “other” (15.87%), Buddhism (5.26%), Islam (4.77%), Hinduism (3.12%), and Judaism (0.39%).

More than half of the participants (58.2%) reported being single, while others reported being divorced (2.04%), widowed (0.58%), or separated (0.1%). The remainder were either married (20.35%), engaged (1.36%), or in a de facto relationship (13.34%). In our sample, 3.7% of the participants reported their marital status as “other”.

### Data Analysis

**Fig. 1:**
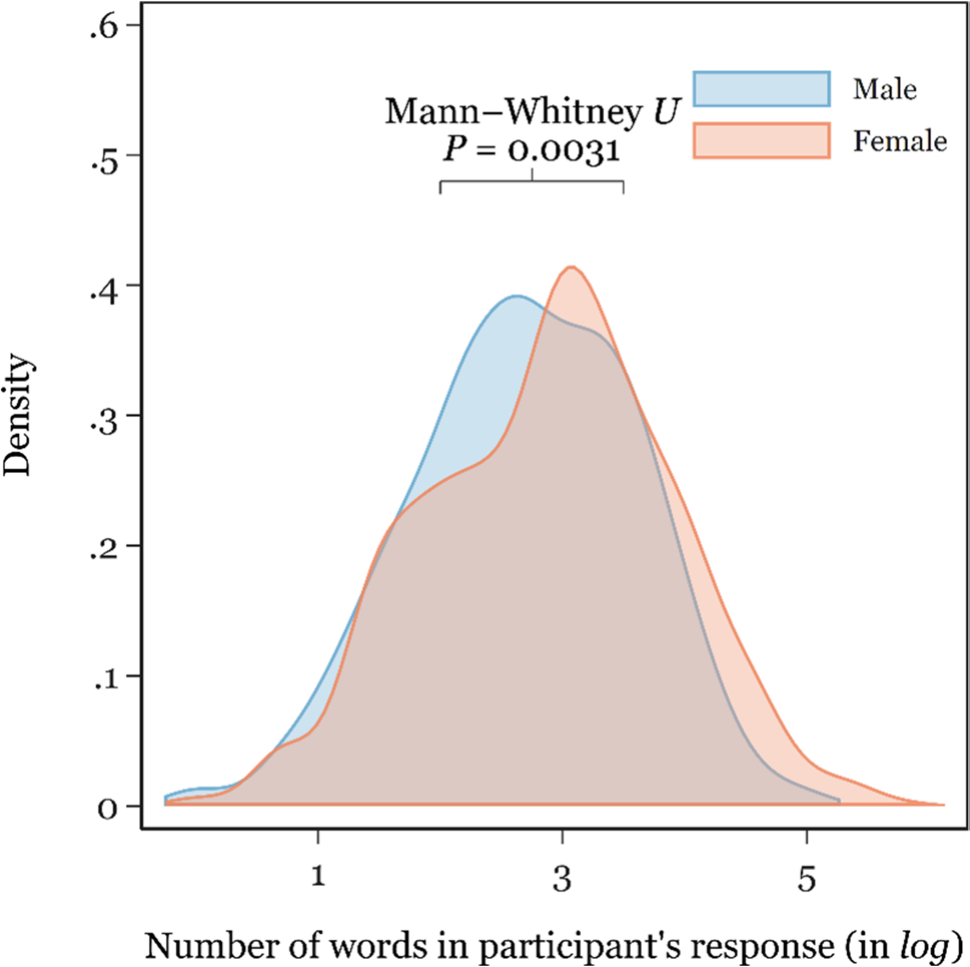
Distribution of the response average word count by participant sex

The written responses female participants provided were (on average) longer (mean = 29.1 words, *SD* = 34.0), compared with the responses provided by males (mean = 21.6 words, *SD* = 20.4). As a visual representation, these data are presented graphically in Fig. 1.

Thematic analysis was selected as the data analysis method as it provides substantial flexibility and adaptability across a wide range of datasets and research aims [42, 43]. Furthermore, it allowed the research team to be instrumental in the identification and interpretation of themes [43]. The researchers followed the broad step-by-step guidelines for thematic content analysis [42] to explore the participants’ reported barriers to gamete donation. This included (i) familiarisation with the data, (ii) generating initial codes and a codebook, (iii) searching for themes, (iv) reviewing themes, and (v) defining and naming themes.

Development of the code book and the final theme categorisation were completed in tandem. This work was completed by two of the authors. Both researchers were postgraduate students at the time and were experienced in using this methodology. The same qualitative methodology has been employed by one of the researchers in another peer-reviewed study (see [44]).

The codebook development stage followed a typical inductive thematic analysis approach [45], attempting to identify as many codes as possible within the raw data. The process sought commonalities among responses to produce commonality between the “voices” of the participants [45]. The data were viewed as a whole in order to identify overlapping perspectives and to avoid being led by key identifiers, such as age or sex. The researchers identified approximately thirty codes during preliminary analysis, which was followed by early transparency and inter-reliability checks of the codebook. Where the codes appeared to be idiosyncratic or include an ambiguous term, they were reconsidered or renamed. These checks also assessed the validity of the inductive approach by checking its relevance to the current literature. For example, the tethered biological connection that participants have with tissue donation has historically been associated with kinship or kin selection. Being that this is an understood construct within the literature, it was deemed appropriate to continue with that as the description. During this stage, we identified a new overarching theme that may not have been completely considered in the current literature: conditional willingness (CW). Saturation was not a consideration for this analysis in the same way as qualitative interviews, as all data was collected via a survey; thus, all data (comments) were coded.

The 1027 participants’ responses were collated in an Excel spreadsheet with key identifiers, such as sex, removed. The data were then read in an *active* way, focusing on asemantic approach (i.e. the explicit or surface meanings of the data; [42]). This allowed for the emergence of precursory ideas.

To ensure consistency across the second stage of analysis, initial codes were generated, with preliminary analyses identifying approximately thirty codes. These codes were then collated into potential themes, highlighting several low-order themes, which then produced several potential high-order themes [42]. It should be noted that the participants’ responses were continuously evaluated as new themes were identified.

We were able to distinguish seven categories that explain CW, where the participant is open to more information and potential future willingness to consider becoming a tissue donor. The categories that contributed to CW were information and education, kin selection and inclusive fitness, control, reproductive history, joint decision-making, incentive or motivation, and willingness. Four categories described under the overarching theme of *Barriers* were ineligible, moral or ethical, socio-cultural or social norm, and religion. *unconsidered* categorises participants’ ambivalent responses that provided neither willingness to consider nor an openness to new information. Finally, *conscientious objector* describes responses reflecting a flat refusal to consider regardless of new information. Importantly, this category was not always directly linked with additional information explained by a barrier, for example, religion.

Data were then evaluated for a final time using the theme and categorisation framework. As shown in Braun and Clarke [42] description of thematic analysis, responses may fall within multiple themes. For example, the following quote from a respondent fits within the themes of unconsidered, willing, and reproductive history with the following quote:

“*I have never thought about donating my gametes. I am not against the process. If anything I would like to ensure that I would be able to have children of my own one day.*”

This thematic content analysis process has illustrated the connectivity of reasons (for example: kinship, age, and need for financial reimbursement) that participants describe when given the opportunity to explain. With this rich data, we gain a greater understanding of participant perspectives that impact their willingness or unwillingness to consider donating their gametes.

A summary table was then produced by conducting an iterative process to arrive at a consensus of four overarching higher-order/core themes and eleven lower order themes. Each overarching theme was then further refined, which allowed for clear definitions and naming of the following core themes: (i) *conditional willingness*, (ii) *barriers*, (iii) *unconsidered*, and (iv) *conscientious objector* (see Table 1).

**Table 1:** Thematic categorisation of responses

Core themes	Lower order themes	Description
*Conditional willingness*	1. Information and education	• The participant had no previous information on donation or education relating to assisted reproductive medicine or gamete donation
	2. Kin selection and Inclusive fitness	• Willing donation for immediate or extended family
	3. Control	• Donation to a known recipient • A known outcome of donation • Directly for co-parenting • Donation for science
	4. Reproductive history	• Possibly willing after completion of one’s own family • Belief that there is a possibility of negative impact (jeopardise) on one’s own fertility • Unsure of one’s own fertility (a question of suitability) • Fertility age (a question of suitability) • Invasive and physical cost
	5. Joint decision-making	• Identification of influence by or consideration, including a spouse, a partner, or a family member
	6. Incentive or motivation	• Financial, ego, altruism, prestige, recognition
	7. Willing	• Willingness to contribute • Willingness to seek information • Willingness to help others
*Barriers*	8. Ineligible	• Vasectomy, hysterectomy, history of IVF or ART, other medical or biological barrier
	9. Morality or ethical	• Moral or ethical issues identified as a barrier
	10. Sociocultural or social norm	• A cultural or social norm identified as a barrier • Feeling that donation of RT is different, strange, weird, uncomfortable, not something that people do
	11. Religion	• Religion identified as a definitive barrier
*Unconsidered*		• Aware of ART/DI but have not considered
*Conscientious objector*		• Refusal based on principle

## Results

### Core Theme 1: Conditional Willingness

While the majority of the participants experienced or stated a current barrier to justify their history of non-donation, they clarified with context that they would consider gamete donation (*conditional willingness*) if the said barrier was removed or a specified requirement was fulfilled. The consensus was not that the participants had strong attitudes either way; rather, that the participants needed something further to assure or assist provision in their future decision-making.

From these responses, we distinguished several lower-order themes of *conditional willingness*. The first lower-order theme is related to *information and education*. More specifically, the participants felt that they had not encountered sufficient relevant gamete donation information and or education in order to donate their gamete. For example, the participants often responded with “*I believe it is something I would consider if I was presented with information that would convince me that it was a good idea*” (female, 20), or “*I want to but don’t know how*” (female, 18).

Another commonly observed example of *conditional willingness* was expressed in the form of *kin selection and inclusive fitness*. This lower-order theme was observed among the participants who expressed that sharing their gametes (or “*biological property,*” as articulated in some cases) with strangers was the biggest barrier relating to donation. For example, one respondent supported their position with, “*This is because my eggs are made of my genetic information and I do not feel comfortable having a stranger walking around with my genetic code*” (female, 19). Others expressed that they thought strangers raising “their” children was a large barrier “*I don*’*t want other couples to have kids from my eggs, I feel like I should be their parent too*” (female, 26); however, in this group, the participants also conveyed they would be willing to share their gametes with immediate and/or extended family, or close friends, only “*I think if a person I knew and trusted or family member couldn*’*t conceive, I would happily donate my eggs*” (female, 33).

C*ontrol* was another lower-order theme, representing a group of participants who felt that the lack of control associated with gamete donation posed the largest barrier. This included the lack of control in knowing to whom the gamete would be donated and the outcome of the donation. Several participants expressed that they would donate only if they could control how the gamete was used. For example, the participants were open to donating gametes for “science” purposes, but not for ART, “*I wouldn’t be opposed to donating for research purposes, but not to create a child*” (male, 22). While others did not feel comfortable not knowing the outcome of the donation, “*I am anxious about the thought that a biological child of mine might grow up without love and protection*” (female, 34), and “*I want to be aware of the amount of children I will leave on this world*” (male, 34). Additionally, some felt that they could not control future outcomes, expressing that they did not want the offspring to contact them in the future (because they/legislation could not currently control this). In the latter group, many participants felt that they would be willing to donate in the future, on the premise they could firmly ensure that they would be kept anonymous to any future offspring, as they were “*concerned about child support implications from donation*” (male, 48).

Another lower-order theme of *conditional willingness* emerged as a function of *reproductive future/history*. Many described their own reproductive concerns as a barrier to gamete donation. For example, some shared the belief that potential negative impacts of the process may jeopardise their own fertility: “*I want to have children in the future and would prefer to keep my eggs for my own use given that I might have reproductive issues *etc.” (female, 21), and that gamete donation is too invasive and costly: “*It’s an intrusive, time-consuming, and [from what I’ve heard] painful experience*” (female, 27). In this lower-order theme, we found that the participants were potentially willing to donate provided they had completed their own family, while others expressed their current specific fertility/reproductive health (namely questions of suitability, e.g. age, pathology) as a barrier to gamete donation.

A less common lower-order theme was *joint decision-making*, which represented the participants who felt a significant influence of or acknowledged the need to include their spouses’, partners’, or family members’ feelings and considerations in any decision to donate. They stated, with qualification, that, if the other party were accepting of their decision, perhaps, they would be likely to donate in the future: “*I would feel an obligation to my future partner/family to make a joint decision*, *especially as it would involve the creation of half brothers or sisters*” (female, 22)*,* and “*Maybe after getting married and with the consent of my husband I might donate my eggs*” (female, 27)*.*

The participants also commonly stated that *incentives and motivation* in gamete donation were conditions for donating. For example, “*I would do it if I received money for it*” (male, 21), and “*I can’t justify undergoing a surgical procedure without any form of compensation*” (female, 18), while others stated feelings similar to “*I would happily be a sperm donor but as it stands I don’t have much motivation to go out of my way to do it*” (male, 19).

Finally, included in our theme of *conditional willingness* is the lower-order theme of *willing*. We found that, while they had not yet donated their gametes, several participants expressed that they were not opposed to doing it and stated their willingness to contribute and help others in the future.

### Core Theme 2: Barriers

Many participants stated barriers to donation. Commonly, these barriers related to objections based on eligibility and/or belief. This theme indicates that many participants provided a strong and conscious decision as to why they have not donated by providing insight into the common barriers experienced.

For example, *ineligibility* was a barrier reported by a number of participants as they explained they had had a vasectomy or hysterectomy, a history of IVF or ART, or other medical conditions or biological barriers that limited their ability to donate.

Some respondents highlighted moral or ethical barriers to donation, namely, that it is “incorrect” to participate in gamete donation: “*I never felt the need of donating eggs. There are millions of kids who don’t have parents, if someone needs a kid, why can’t they give a life to those poor kids*” (female, 28), and “*I do not think it is appropriate to have a biological child from other parents*” (male, 26).

Other barriers included *sociocultural or social norms*. These participants felt that donating was not viewed favourably among their peers, or within society. For example, some participants were very direct in stating that they felt that gamete donation was “*a weird pressure for the rest of my life*” (male, 27)*,* “*not something that we often hear about*” (male, 29), and that, “*donation is kinda taboo, which is not a right thing*” (male, 27).

Similarly, others expressed that donation conflicts with their *religious beliefs* “*This also go against my religious perspective*” (female, 32), and that they “*Do not agree with this practice, and my religion forbids it*” (male, 23).

### Core Theme 3: Unconsidered

Some participants indicated that they held an ambivalent position on gamete donation. This *unconsidered* view on donation was common among participants. Several reported that, while they were well aware of ART and the prospect of donation, they had never been cognitively engaged or positioned to make a decision as to whether they would donate or not “*I’ve never really thought about it*” (female, 20), and “*I have never thought about doing it, it hasn’t crossed my mind as an option for donation*” (female, 28). Others had never considered donating because they had never been approached to do so “*Nobody asked me*” (male, 33).

### Core Theme 4: Conscientious Objector

Finally, *conscientious objector* referred to a firm refusal to participate in gamete donation. This overarching theme did not always include an explicit barrier to donating (i.e. “*I do not want to do it*” (female, 33), and “*There is no way I would do this in a million years*” (female, 55). Rather, it was commonly expressed as an outright refusal regardless of new information, incentive, or medical and societal demand for gametes. This theme was the least common.

### Sex Differences in Responses

In Table 2, we present the two-sample test of proportions to explore differences between male and female core and lower-order themes for not yet donating.

**Table 2:** A two-sample test of proportions

Proportion	Female (%)	Male (%)	Diff	*z*	*p*-val
Conditional willingness	70.0	61.8	8.2	2.65**	0.008
* Information and education*	22.5	26.9	− 4.4	− 1.57	0.117
* Kin selection and inclusive fitness*	13.8	8.1	5.7	2.67**	0.008
* Control*	19.2	23.1	− 3.9	− 1.46	0.145
* Reproductive history*	33.5	12.1	21.3	7.34***	0.000
* Joint decision*-*making*	1.2	1.2	0	0.03	0.979
* Incentive or motivation*	2.3	2.9	− 0.5	− 0.52	0.602
* Willing*	12.8	11.6	1.2	0.56	0.576
Barriers	17.3	23.7	− 6.4	− 2.44*	0.015
* Ineligible*	5.6	4.0	1.5	1.06	0.289
* Morality or ethics*	8.1	8.7	− 0.6	− 0.33	0.744
* Socio-cultural or social norm*	3.2	9.2	− 6.0	− 4.08***	0.000
* Religion*	0.9	2.6	− 1.7	− 2.17*	0.030
Unconsidered	37.4	34.1	3.3	1.05	0.293
Conscientious objector	11.2	15.0	− 3.9	− 1.77†	0.076
Observations	681	346			

***, **, *, and † denote significance at the 0.1, 1, 5, and 10% levels, respectively

Females were significantly more likely than males to state some form of *conditional willingness* (8.2% difference; *p* = 0.008), and males were more likely than females to state some form of *barrier* (6.4%; *p* = 0.015) or *conscientious objection* (3.9% difference; *p* = 0.076) in their response.

For the lower-order themes, there were statistically significant differences with females being more likely than males to justify their non-donation using content, reflecting their *reproductive history* (21.3% difference; *p* = 0.000) or *kin selection and inclusive fitness* (5.7% difference; *p* = 0.008). On the other hand, the males were more likely than the females to explain their non-donation by using *sociocultural or social norms* (6% difference; *p* = 0.000) or *religion* (1.7% difference; *p* = 0.030) as justification.

## Discussion

As the majority of the population will always be non-donors, RT donation education and awareness in non-donor populations are critical for increasing future donation numbers [21]. By identifying key themes in the preferences and behaviour of the general population who have not yet donated, studies such as this are an integral part of understanding how to engage and motivate such non-donor populations.

Our thematic content analysis illustrates the breadth of reasons and justifications people offer when given the opportunity to explain their non-donation decision-making. Our study provides novel findings on the factors that influence non-donors’ preferences and perspectives, particularly concerns regarding perceived barriers. Interestingly, we find that women appear more willing (in principle) to participate in RT donation than men and are more likely to provide reasons for not donating that relate to their own fertility and own biological families. These results compare with a recent study by Bracewell-Milnes et al. [46] of some 600 UK women, who, in majority, were in approval of egg donation but stated “*little or no prior knowledge*” of egg donation as a barrier (p. 2189). And, similarly, women’s biological age was a major consideration (see also [48]).

Conversely, we find that men are more likely to state an explicit barrier to their participation, or provide specific examples of religious, cultural, or social norms preventing their participation. Recent research from Portugal (*n* = 282) has shown that, for men, religious values appear negatively correlated with motivations and attitudes to sperm donation [49]. Future research would thus do well to explore exactly why such cultural norms (in the context of RT donation) appear to be so negatively correlated for men and yet so positively for women.

The authors note the current study is not without limitations. Firstly, thematic categorisation of more than 1000 written responses by the research team is, in some part, subjective. That said, the research team employed a stringent and specific theoretical framework for such thematic content analysis [42]. Secondly, data collected in this study comes from an online sample of self-selected participants and, as such, is a convenience sample. For that reason, it is unclear whether the individuals participating in the current study are completely representative of the broader population, which is not just an issue for the current study but is a broader and ongoing methodological concern for all behavioural science research. That said, this is a commonly used method of data capture in the field of gamete donation research [46].

Understanding non-donor preferences and barriers to gamete donation is critical in informing targeted interventions aimed at future potential donors. That so many of our study’sparticipants reported in-principal willingness for future participation in RT donation speaks to the need for increased research in the non-donor space.

## Data Availability

Data are available from the corresponding author upon request.

## Code availability

Codes are available from the corresponding author upon request.
